# The British Hospitals Association

**Published:** 1916-09-02

**Authors:** 


### The British Hospitals Association.

### A NEW DEPARTURE DURING WAR-TIME.

An announcement, signed by the chairman and hon. secretaries, has been issued by this Association, intimating that it is proposed to issue reports on subjects relating to work belonging to hospitals from time to time, and the first of such reports, prepared by the Executive Committee at the request of the Council, is circulated with this notice. The brochure is entitled "Report of the Executive Committee, August 1916." It contains a list of the vice-presidents, Council, and officers of the Association, and sets forth its objects. The report follows these official statements and extends to some sixteen crown octavo pages. It sets forth its object to be to draw the attention of members of the Association to matters of moment as they arise, and proceeds tersely to "indicate several questions which are of great importance to hospitals at the moment." These include correspondence and notices issued by the War Office in relation to the urgent need which exists for medical men for service with the Army. It sets forth the objects of the College of Nursing, and the authors of the report show a clear leaning against the registration of nurses, and an imperfect understanding of the Hon. Arthur Stanley's speeches and explanations on the position of the College of Nursing to-day, and of the practical agreement amongst those interested in, or responsible for, the training of nurses and the presentation to Parliament in the ensuing autumn of an agreed Bill granting its sanction to the setting up of a register of nurses under State authority. Dissatisfaction is expressed with the proposal that the voluntary hospitals should nominate two representatives to serve upon the Consultative Board of the College of Nursing, and the authors seem to be unaware that out of forty-five members of the Council of the College a proportion are to be representatives of the nurse-training schools.

For the rest, the report deals mainly with Government schemes and Local Government Board action with regard to certain diseases. the maternal and infantile welfare scheme, and public-health measures. The latter includes overcrowding, the insanitary condition of poorer homes, and public health, and records the opinion that it would be in the best interests of most hospitals to consider carefully to what extent they can fall in with the proposals of the Local Government Board, even if the work the hospitals can immediately undertake should prove to be strictly limited in extent. The extern department, out-patient treatment, the elimination of trivial cases, and the National Insurance Acts are also touched upon. In conclusion, the report asks for suggestions of ways in which the Association may be most useful to the members, and is signed by the chairman and the members of the Executive Committee.

The brochure is presented in an attractive form, is well printed, and its arrangement and general get-up reflect credit on the printers and the Executive Committee.

